# Outcomes of pregnant patients with low back pain undergoing chiropractic treatment: a prospective cohort study with short term, medium term and 1 year follow-up

**DOI:** 10.1186/2045-709X-22-15

**Published:** 2014-04-01

**Authors:** Cynthia K Peterson, Daniel Mühlemann, Barry Kim Humphreys

**Affiliations:** 1 Department of Chiropractic Medicine, Orthopaedic University Hospital Balgrist, University of Zürich, Forchstrasse 340, Zürich, Switzerland

**Keywords:** Pregnancy, Low back pain, Pelvic pain, Outcomes, Chiropractic, Treatment

## Abstract

**Background:**

Low back pain in pregnancy is common and research evidence on the response to chiropractic treatment is limited. The purposes of this study are 1) to report outcomes in pregnant patients receiving chiropractic treatment; 2) to compare outcomes from subgroups; 3) to assess predictors of outcome.

**Methods:**

Pregnant patients with low back or pelvic pain, no contraindications to manipulative therapy and no manual therapy in the prior 3 months were recruited.

Baseline numerical rating scale (NRS) and Oswestry questionnaire data were collected. Duration of complaint, number of previous LBP episodes, LBP during a previous pregnancy, and category of pain location were recorded.

The patient’s global impression of change (PGIC) (primary outcome), NRS, and Oswestry data (secondary outcomes) were collected at 1 week, 1 and 3 months after the first treatment. At 6 months and 1 year the PGIC and NRS scores were collected. PGIC responses of ‘better’ or ‘much better’ were categorized as ‘improved’.

The proportion of patients ‘improved’ at each time point was calculated. Chi-squared test compared subgroups with ‘improvement’. Baseline and follow-up NRS and Oswestry scores were compared using the paired t-test. The unpaired t-test compared NRS and Oswestry scores in patients with and without a history of LBP and with and without LBP during a previous pregnancy. Anova compared baseline and follow-up NRS and Oswestry scores by pain location category and category of number of previous LBP episodes. Logistic regression analysis also was also performed.

**Results:**

52% of 115 recruited patients ‘improved’ at 1 week, 70% at 1 month, 85% at 3 months, 90% at 6 months and 88% at 1 year. There were significant reductions in NRS and Oswestry scores (p < 0.0005). Category of previous LBP episodes number at one year (p = 0.02) was related to ,improvement’ when analyzed alone, but was not strongly predictive in logistic regression. Patients with more prior LBP episodes had higher 1 year NRS scores (p = 0.013).

**Conclusions:**

Most pregnant patients undergoing chiropractic treatment reported clinically relevant improvement at all time points. No single variable was strongly predictive of, improvement’ in the logistic regression model.

## Background

Low back and pelvic pain in pregnant women is such a common phenomenon that it is often considered a normal part of the pregnancy [1-3]. However, the high prevalence of this problem (50-80% of women) and the impact that this may have on their quality of life, as well as the fact that back pain during pregnancy is commonly linked to low back pain persisting after pregnancy, mandates that it be taken seriously by health care practitioners [1-6]. Many of these patients rate their back pain as moderate to severe with a small percentage claiming to be significantly disabled by the pain [6-8].

Pregnancy-related low back pain is most often divided into 3 categories based on location. These are: lumbar spine pain (LP), posterior pelvic pain (PPP), or a combination of these two [1,2,9], with posterior pelvic pain reported to be the most common presentation [1,10] and the location most specific for pregnant patients [9]. However, other authors have used 4 categories for pelvic only pain, including anterior pain at the pubic symphysis (symphysiolysis) but excluding lumbar spine pain [11].

Although the etiology of low back pain associated with pregnancy is not definitively known, the predominate theories include biomechanical changes due to the enlarging uterus resulting in an increasing lumbar lordosis and the influence of the hormone relaxin on stabilizing ligaments leading to hypermobility of joints [12-14].

The treatment of pregnancy-related low back pain has often been a ‘watch and wait’ approach, as medication use in pregnancy is strongly discouraged in many cases [15] and it is assumed that the pain will disappear after delivery. Two recent systematic reviews of the literature looked specifically at the evidence for chiropractic treatment of pregnancy-related low back pain and both reviews concluded that there is research evidence demonstrating that chiropractic care is associated with improved outcomes, but that the evidence is limited due to the low to moderate quality of the studies [4,6]. A prospective cohort outcomes study on pregnant patients with low back pain receiving chiropractic treatment from a single practitioner published since the systematic reviews evaluated data from 78 patients [16]. Seventy three percent of that cohort reported ‘good’ or ‘excellent’ outcomes from chiropractic treatment performed by a single chiropractor and his physical therapist. However, the outcomes varied depending upon the specific location of the pain.

Finally a prospective randomized trial was published in 2013 which compared routine obstetrical care for pregnant patients suffering from low back pain with chiropractic treatment consisting of manual therapy, stabilization exercises and patient education [17]. The chiropractic treatment group reported statistically and clinically significant improvement compared to patients in the routine obstetrical care group. However, outcome data was only collected between 5 and 9 weeks after the baseline data and prior to delivery of the baby.

Additional research is therefore needed from a larger number of chiropractors to assess outcomes as would be obtained in routine chiropractic practice and to follow the patients for a longer time period. Furthermore, it is desirable to see if factors associated with improvement or failure to improve identified in previous studies will also be found in this current study [16]. Thus, the purposes of this study are 1) to report outcomes at various time points up to 1 year in pregnant patients undergoing chiropractic treatment for low back and pelvic pain; 2) to compare outcomes from various subgroups of patients; and 3 to investigate the various demographic factors as predictors out outcome.

## Methods

This is a prospective, cohort, outcomes study. Patients with baseline and 1 year data are included in the study. Cantonal ethics approval was obtained prior to the start of the study and all patients signed informed consent.

Pregnant patients over the age of 18 with low back pain, pelvic pain, or both of any duration who have not undergone chiropractic or manual therapy in the prior 3 months were recruited from chiropractic practices in Switzerland. All chiropractors in the German and French speaking regions of the country were invited to submit patients but 2 multi-clinician practices in particular that receive a high number of referrals from gyaecologists were targeted specifically. These two practices were asked to recruit all consenting pregnant patients. Patients with specific pathologies of the lumbar spine that are contraindications to chiropractic manipulative treatment, such as tumors, infections, inflammatory spondylarthropathies, acute fractures, Paget’s disease and severe osteoporosis, were excluded.

Chiropractic treatment was not standardized to any specific treatment methods or frequencies and was left to the discretion of the treating clinician. However, it is known from the Swiss job analysis study that the Diversified method of high velocity, low amplitude spinal manipulative therapy (SMT) is the most common SMT method used in this country [18].

### Baseline data and outcome measures

The numerical rating scale (NRS) for pain, where 0 = no pain and 10 = the worst pain imaginable, and the Oswestry questionnaire, which has been validated in German and French [19,20] were administered to the patient immediately prior to the first treatment by the office staff of the practice. Additional information provided by the treating chiropractor at the initial consultation included: patient age, gestational week, number of previous pregnancies, work status, whether or not the patient smokes, duration of current complaint, number of previous LBP episodes, LBP during a previous pregnancy (yes/no), location category of pain (lumbar pain only (LP), pelvic pain only (PP), both areas) and exercise level (regular, occasional, none). The number of previous episodes of LBP was further categorized into one of three groups: None, 1 – 4 episodes, ≥ 5 episodes.

One week after the first consultation/treatment, data from the NRS, patient’s global impression of change (PGIC) scale, and the Oswestry questionnaire were collected from the patient via a short telephone interview. Similarly, these same data were collected at 1 month and 3 months after the start of chiropractic treatment via telephone interviews. At 6 months and 1 year after the first chiropractic treatment, the NRS and PGIC scores were collected, but not the Oswestry data. These telephone interviews were conducted by trained research assistants at the university, unknown to the patients or clinicians. The PGIC scale was a 7 point verbal scale, including the options ‘much worse’, ‘worse’, ‘slightly worse’, ‘no change’, ‘slightly better’, ‘better’, and ‘much better’. Patients responding ‘better’ or ‘much better’ were categorized as ‘improved’ and all other patients as ‘not improved’. This was considered the primary outcome measure. Additionally, one question was included on the 1 year follow-up data collection concerning the patient satisfaction with their treatment. The options included: ‘very happy’, ‘happy’, ‘neutral’, ‘unhappy’ and ‘very unhappy’.

The time frame within which each data collection telephone interview was allowed was narrow. If a patient could not be reached within the window of time allowed, that data collection time point was missed but the patient remained in the study. However, if 3 consecutive data collection telephone interviews were missed, the patient was deleted from the study.

### Statistical methods

Descriptive statistics were calculated. The proportion (%) of patients improved at each data collection time point was calculated. Subgroup analysis was carried out using ‘improved’ or ‘not improved’ as categorical variables. The chi-squared test was used to compare the presence/absence of LBP during a previous pregnancy, category of the number of previous episodes of LBP, and the category of the location of LBP with ‘improvement’. The scores on the baseline and follow-up NRS for pain and Oswestry questionnaire were compared using the paired t-test. The unpaired t-test was used to compare NRS and Oswestry scores in patients with and without a prior history of LBP and for patients with and without LBP during a previous pregnancy. Anova was used to assess for differences in baseline and follow-up NRS and Oswestry scores by location category of the LBP and category of the number of previous episodes of LBP. NRS and Oswestry change scores were also calculated for each follow-up time period compared to the baseline scores.

Logistic regression analysis was also performed to determine statistically significant predictors of improvement using the 10 baseline variables (age, number of previous pregnancies, chronicity category, history of LBP, LBP during a previous pregnancy, gestational week, location category for LBP, work status, exercise level category, smoking status) at 1 month, 3 months, 6 months and 1 year after start of treatment. The full model containing all predictors was also assessed for statistical significance and ability to distinguish between patients who improved or did not improve as well as the proportion of correctly classified cases.

## Results

Baseline and 1 year data were available on 115 patients. In order to obtain 1 year outcomes data on these 115 patients, 143 were enrolled with baseline data. Seven of these 143 patients requested to drop out of the study prior to the 1 year data collection time point and 28 other patients were not able to be contacted for 3 consecutive telephone data collection time periods. Thus they were deleted from the study. The patients came from a total of 15 different chiropractors. One hundred of the 115 patients came from the 2 practices specifically targeted. One of these sites has 4 chiropractors and the other 3 and all referred pregnant patients to the study.

The mean patient age was 32.96 (SD = 4.64) years and the mean gestational week was 26.21 (SD = 6.98). Table 1 shows the results of the various baseline demographic factors. A slight majority of patients (53.1%) were in their 3^rd^ trimester of pregnancy (Table 1) and thus would have delivered their babies by the 3 month follow-up time point. There was a fairly even distribution in the percentage of patients complaining of lumbar pain only, pelvic pain only or a combination of the two areas. Of the 53 patients who had at least one previous pregnancy, 31 (58%) reported having experienced back pain during the previous pregnancies.

**Table 1:** Percentage of patients with the various baseline demographic factors

**Baseline variable**				
**Previous Pregnancy number**	None = 51.3%	1 = 35.4%	2 = 12.4%	3 = 0.9%
	(N = 59)	(N = 41)	(N = 14)	(N = 1)
**Chronicity LBP**	0-4 Weeks = 58.3%	4-12 Weeks = 30.4%	More than 12 Weeks = 11.3%	
	(N = 67)	(N = 35)	(N = 13)	
**History of LBP**	No = 46%	Yes = 54%		
	(N = 53)	(N = 62)		
**Number of Prior LBP episodes**	None = 36.5%	1 – 3 = 27.0%	4 or more = 36.5%	
	(N = 42)	(N = 31)	(N = 42)	
**LBP during previous pregnancy**	No previous pregnancy = 53.0%	Yes = 27.0%	No = 20.0%	
	(N = 61)	N = 31)	(N = 23)	
**Location of LBP**	LBP only = 32.2%	Pelvic pain only = 31.3%	Combination = 35.7%	
	(N = 37)	(N = 36)	(N = 41)	
**Work Status**	Working = 71.3%	Off due to LBP = 10.4%	Not working = 18.3%	
	(N = 82)	(N = 12)	(N = 21)	
**Smoker**	No = 96.5%	Yes = 3.5%		
	(N = 111)	(N = 4)		
**Exercise**	Regular = 58.5%	Occasional = 18.4%	None = 22.8%	
	(N = 68)	(N = 21)	(N = 26)	
**Gestational Week Category**	1^st^ Trimester = 5.3%	2^nd^ Trimester = 41.6%	3^rd^ Trimester = 53.1%	
	N = 6)	(N = 48)	(N = 61)	

*LBP* low back pain, *N* number or patients.

Over half of the patients reported clinically relevant ‘improvement’ at 1 week, with the vast majority ‘improved’ at all subsequent data collection time points (Table 2). Statistically significant reductions in NRS and Oswestry scores (p < 0.0005) at all follow-up time points compared to the baseline scores was also noted (Table 2). Comparing the category of the number of previous episodes of LBP with ‘improvement’ found a significant relationship at 1 week (p = 0.035) and especially at 1 year (p = 0.02). Patients who ‘improve’ have fewer episodes of prior LBP. Patients with more previous episodes (≥ 5, n = 29) of LBP also had significantly higher 1 year NRS scores with a mean of 2.16 (SD = 2.36) compared to a score of 0.60 (SD = 1.07) for patients with no prior LBP history (n = 30) (p = 0.013). Patients who reported having LBP in a previous pregnancy had significantly lower baseline NRS scores (5.22, SD = 2.10) compared to those who did not have LBP in a previous pregnancy (6.35, SD = 1.84) (p = 0.01). However, there was no significant difference in the NRS scores between these two groups at any follow-up time point. There was no significant link between the category of location of LBP (i.e. low back pain only, pelvic pain only, or both) and ‘improvement’ at any of the follow-up time points.

**Table 2:** Baseline and outcome data for all patients at the various time points

	**Baseline data (115 PTS)**	**1 week (100 PTS)**	**1 month (106 PTS)**	**3 months (104 PTS)**	**6 months (110 PTS)**	**1 year (115 PTS)**
***PGIC***		*52*% *= Much better or better*	*70*% *= Much better or better*	*85*% *= Much better or better*	*90*% *=* *Much better or better*	*88*% *=* *Much better or better*
		*6*% *= worse*	*8*% *= worse*	*3*% *= worse*	*4*% *= worse*	*1*% *= slightly worse*
**NRS (Mean (SD))**	6.07 (1.91)	4.22* (2.13)	3.06* (2.63)	1.58* (2.15)	1.10* (2.0)	1.19* (1.86)
**NRS Change (Mean (SD))**		1.85 (2.32)	3.06 (3.27)	4.54 (2.85)	5.07 (2.78)	4.92 (2.59)
**OSWESTRY (Mean (SD))**	14.33 (8.01)	11.48* (7.27)	8.36* (7.18)	4.97* (6.84)		
**Oswestry Change (Mean (SD))**		2.85 (5.79)	6.36 (8.35)	9.70 (10.29)		

*NRS* numerical rating scale for pain, *PGIC* patient’s global impression of change, *SD* Standard deviation, *PTS* patients.

* = p < 0.0001 compared to baseline score.

At 1 year, 85.2% of patients (n = 98) were ‘very happy’ or ‘happy’ with their chiropractic treatment and 6% (n = 7) were ‘unhappy’.

Logistic regression analysis using the 10 independent variables (Table 1) compared to the primary outcome of, improvement‘ showed that the full model was statistically significant, *X*^2^ (5, N = 115) = 40.71, p < 0.009 at 1 month, indicating that the model could distinguish between patients ‘improved’ and ‘not improved’. Between 55.7% and 79.0% of the variability is explained by this set of variables at 1 month after start of treatment. However, no single independent variable made a unique statistically significant contribution to the model. Additionally, at 3 months, 6 months and 1 year the model was not statistically significant as the vast majority of patients were ‘improved’.

## Discussion

The results of this current study which showed that a high proportion of pregnant patients with LBP undergoing chiropractic treatment reported clinically relevant ‘improvement’ support those published in a recent cohort study as well as the recent randomized clinical trial (RCT) looking at chiropractic treatment for pregnant patients with low back or pelvic pain [16,17]. The most recent systematic reviews of the literature for interventions for preventing and treating pelvic and back pain in pregnancy [5] and more specifically chiropractic treatment for these patients [4,6] unanimously concluded that the available research evidence at that time was low to moderate quality at best. However, these reviews were published prior to the cohort study by Murphy et al. published in 2009 [16] and the excellent RCT by George et al. published in 2013 [17].

In the RCT by George et al. one treatment arm of the trial on pregnant women suffering from LB, PP or both included treatment by a chiropractor. The results clearly showed a statistically and clinically significant greater level of improvement for the patients who received the additional chiropractic treatment [17]. However, outcomes in that RCT were only measured at one time point between 5 and 9 weeks after the start of treatment and while the patients were still pregnant whereas this current study measured outcomes at 5 different and consistent time points during the pregnancy and after delivery. Comparing the baseline scores between this current cohort study with the baseline scores for the patients in the RCT who were treated with chiropractic shows that they were nearly identical, including the standard deviations (5.8 +/- 2.2 in the RCT, 6.07 +/- 1.91 in this current study). Additionally, the mean gestational weeks were also nearly identical in the two studies. Comparing the 1 month NRS pain scores in this current study with the NRS scores in the RCT measured between 5 and 9 weeks after the start of treatment for those patients in the treatment arm that received chiropractic treatment also shows that they are nearly identical. The NRS change score in the RCT was 2.9 and in this cohort study it was 3.06 [17]. When looking at the mean NRS score at 3 months after the first treatment in this current study it is less than 1/3 of the original baseline score. However, approximately half of the patients would have delivered their babies by this 3 month data collection time point and this likely had a positive impact on this outcome. Therefore, although this cohort study is not a randomized clinical trial and thus the outcomes cannot be attributed to the chiropractic treatment, the strong similarity between these results and those from the recently published RCT supports their validity.

Comparing the outcomes from this study on Swiss patients with those from a very similarly designed outcomes study on patients in the United States (US) [16], shows that they are also quite similar. At 1 month after the start of treatment 70% of the patients in this current study reported clinically relevant improvement of ‘much better’ or ‘better’ compared to 73% of patients in the Murphy et al. [16] study. However, the precise time of their outcome data collection is not specified. It was done at the end of active treatment, and this time frame varied between patients. By 3 months after the start of treatment 85% of the patients in this current study reported being clinically significantly improved. It is important to point out that the option ‘slightly better’ on the PGIC scale was not considered clinically relevant improvement in this current study and these patients were classified as unchanged. Unfortunately it is not possible to compare disability scores or other quality of life factors between these two studies because the Murphy et al. paper used the Bournemouth questionnaire [16] whereas our current study used the Oswestry pain and disability questionnaire. The Bournemouth questionnaire would have been a better choice as an outcome measure for these types of patients as it measures more relevant domains, including psychosocial factors, compared to the Oswestry questionnaire. However, at the time that this current study was conducted the Bournemouth questionnaire was not yet translated and validated into German so could not be used whereas the Oswestry questionnaire had been translated and validated into both German and French.

Another relevant, although perhaps not surprising finding from this current cohort study includes the fact that patients who reported a higher number of previous LBP episodes (≥ 5) were less likely to report clinically relevant improvement, particularly at 1 year after the first treatment. However, this factor was not predictive in the logistic regression model. This is consistent with other studies that show that back pain is commonly recurrent [21-23]. However, the location category of the LBP (LB, PP, both) in this current study was not linked with the likelihood of improvement at any data collection time point nor in the logistic regression model. This is different from what Murphy et al. [16] found. They reported that patients with pain in both areas were significantly less likely to report clinically significant improvement in disability, at least in the short-term, compared to the other two groups. Furthermore, the majority of patients in the Murphy et al. [16] study had posterior pelvic pain (58.3%) whereas the location of the LBP in this current Swiss study was quite evenly distributed between the three locations, with approximately 1/3 of the patients reporting pelvic pain. Another difference between the Murphy et al. [16] study and this current one is that Swiss patients with a history of LBP during a previous pregnancy did not have worse outcomes compared to patients without LBP in a prior pregnancy whereas US patients without LBP in a previous pregnancy were more likely to report improvement compared to US patients with LBP in a previous pregnancy. Reasons for the differences in these outcomes between US and Swiss patients can only be speculated upon. Obesity is much more common in the US than in Switzerland and this may result in a higher proportion of women experiencing both LBP and pelvic pain during pregnancy as this could further increase the lumbar lordosis and place additional stresses on the pelvic joints [24].

The literature states that 94% of women who have LBP in a previous pregnancy have recurrent pain with subsequent pregnancies [23]. However, in this current cohort study only 58% of the patients who had experienced a previous pregnancy reported that they had suffered from back pain at that time. The reason for this discrepancy in proportions is unclear. It is also somewhat surprising that patients with more episodes of previous LBP have worse outcomes at the 1 week and 1 year time points but not at the 1, 3 and 6 month time points. Obviously at the 1 year data collection point all patients would have delivered their babies several months before and would likely then fall into the category of ‘usual’ low back pain patients where recurrence is not unusual.

No serious adverse events were reported in this study and over 85% of the patients were happy or very happy with their chiropractic treatment. Adverse events from spinal manipulation to pregnant women or those in the early post-partum period are very rare with only 7 cases found in the literature [25]. All seemed to be related to treating the cervical spine rather than the low back however, and the practitioners involved included chiropractors, a physiotherapist, and a general medical practitioner. A recent qualitative study evaluating the treatment experience of pregnant women under chiropractic care reported that chiropractors’ approach to these patients is patient-centered rather than symptom centered [26]. This may explain why such a high percentage of the patients in this current study were happy with their treatment.

Some of the limitations to this study have been alluded to above. Because this is a cohort study without a control group or other treatment group for comparison, the outcomes reported cannot be assumed to arise from the treatment. Additionally, the patients in this study were treated primarily at two practice sites but by different chiropractors and the details of the types of treatments and treatment dosage are not known. No attempt was made to compare outcomes by practitioner or style of treatment. This would be interesting for future studies. As previously stated, the use of the Oswestry questionnaire was not the best choice for this patient population, but was the best available that was translated and validated in both German and French. Additionally, the assessment of symphysis pubis pain in these pregnant patients was not specifically assessed as it was desired to use those same categories of low back pain in pregnancy that previous papers used in order to make direct comparisons.

## Conclusions

A large proportion of pregnant patients with LBP or pelvic pain undergoing chiropractic treatment report clinically relevant improvement in their symptoms at all time points up to 1 year. There is a relationship between the number of prior episodes of LBP and ‘improvement’ with patients reporting more episodes being less likely to improve. However, this was not a strong predictor of, improvement‘ when placed into a logistic regression model.

## Abbreviations

LBP: Low back pain; LP: Lumbar spine pain; n: Number; NRS: Numeric rating scale for pain; PGIC: Patient’s global impression of change; PPP: Posterior pelvic pain; PTS: Patients; SD: Standard deviation; SMT: Spinal manipulative therapy.

## Competing interests

The authors declare that they have no competing interests.

## Authors’ contributions

CP: Ethics approval submission, Literature search, data entry, analysis and interpretation of data, drafting and revising the manuscript, final approval of the manuscript. DM: Patient recruitment, baseline data collection, patient treatment, final approval of the manuscript. BKH: Concept and design of the study, manuscript preparation and final approval of the manuscript.
